# Implant-supported prosthesis under progressive loading protocol stimulates alveolar bone growth in patients with severe alveolar bone atrophy. Retrospective case series

**DOI:** 10.3389/fdmed.2024.1465137

**Published:** 2024-12-11

**Authors:** Eduardo Anitua, Laura Piñas, Mohammad H. Alkhraisat

**Affiliations:** ^1^ University Institute for Regenerative Medicine and Oral Implantology—UIRMI (UPV/EHU-Fundación Eduardo Anitua), Vitoria, Spain; ^2^ Regenerative Medicine Department, BTI Biotechnology Institute, Vitoria, Spain

**Keywords:** alveolar bone atrophy, mandible, completely edentulous, implant supported complete denture, progressive loading

## Abstract

**Introduction:**

The jaw with severe bone atrophy is a difficult challenge when rehabilitating with dental implants. To be able to place dental implants in the most severe cases and to achieve an increase in bone volume by means of the tension transmitted by the load is a novelty. This work provides data on the alveolar bone changes in a severely atrophic mandible that has been treated with implant supported prosthesis under progressive loading protocol.

**Material and methods:**

This study reported on 3 patients with completely edentulous mandible. In all cases, implants were inserted in the anterior region of the mandible and progressive loading was carried out with an increase in the distal cantilever. The length of the cantilever extension was adapted to growth of the residual alveolar bone at the mandible body. The increase in bone height was controlled in the area of implant placement as well as in the area distal to the implants (1 cm behind the last implant).

**Results:**

This case series described 3 patients where 13 implants were placed. The patients were followed for 17, 19 and 20 years after implants insertion. The mean mandibular residual height of the alveolar bone was 7.8 ± 2.7 mm at the implant site. The mean mandibular body height at 1.0 cm distal to the last implant was 7.0 ± 3.9 mm in the third quadrant and 8.1 ± 4.4 mm in the fourth quadrant. The mean height at the last follow-up was 11.0 ± 3.2 mm (±3.2) in the third quadrant and 11.20 ± 4.4 mm in the fourth quadrant.

**Conclusions:**

Implant-supported prosthesis and progressive loading have resulted in vertical bone growth in a series of patients with extreme atrophy of the mandible. The long-term follow-up indicated that bone growth is confined to the dental implants but has been extended to distant regions resulting in the thickening of the mandibular body and the creation of the absent mandibular canal.

## Introduction

Alveolar bone atrophy, after tooth loss, is cumulative, progressive and irreversible (1–3). Once the teeth are lost, the alveolar bone that depends exclusively on their maintenance is also lost, leaving a basal bone, very cortical, which also undergoes resorption, but much more slowly than the alveolar process (1–3). The mandible is more vulnerable to alveolar bone atrophy than the maxilla (3–5). Completely edentulous mandible with removal prosthesis can lose 21% of the alveolar bone at three months, 36% at six months, and 44% at 12 months (3, 4). Over a period of 25 years, the mandible can lose up to 10–12 mm in height, resulting in the surfacing of the inferior alveolar nerve (5, 6). In the maxilla, the height loss is slower, and may even be half of that in the mandible (2–5). Generally, the rate of alveolar bone resorption has been higher in those patients that lost their teeth more recently (7).

Several anatomical factors predispose the mandible toward more bone loss: higher cortical bone, the insertion of muscles and the transmission of forces from the perioral soft tissues (2). These patterns can also be affected by other co-factors such as age, bone density, gender and the presence of pathologies affecting bone mineralization (8).

The loss of teeth imposes changes in mechanical force transduction to bone. Alsaggaft et al. have shown that the alveolar bone atrophy in the maxilla and the mandible has been significantly higher in patients wearing denture (≥5 years) than those who did not (6). The authors have also observed that severe resorption cases have been mostly observed in the denture wearers group (6). However, the placement of dental implants to support an overdenture have significantly influenced the amount of alveolar bone loss. Şirin et al. (9) have observed that the alveolar bone loss has been significantly lower in patients with implant-supported overdenture than those wearing conventional complete denture. Furthermore, the quality of life is generally superior in patients treated with implant-supported overdenture (IOD) than patients treated with complete denture (CD). In a meta-analysis, Moreno et al. have found statistically significant differences in favor of IOD in stability, speech, comfort and overall patients' satisfaction (10). Gjengedal et al. have reported in a randomized clinical trial that patients with IOD have significantly better ability to chew, less avoidance of some food items and greater willingness to eat more food items (11).

For placing dental implants, sufficient bone quantity should be available/created to ensure long-term stable outcomes. Different bone augmentation procedures are available to treat alveolar bone atrophy. Generally, the incidence of healing complications has been higher as the bone gain is higher (12). This is an important factor to be considered in the treatment of patients with severe alveolar bone atrophy and health status concerns. In such cases, patient treatment is challenging due to the presence of inferior alveolar nerve and its mental branch, lacking of keratinized mucosa and the presence of limited contact area with the denture base (12, 13).

The use of short implants (<10 mm) (14) in completely edentulous patients is a reliable treatment alternative to bone augmentation procedures (15–18). This is supported by similar implant and prosthesis survival rates, less biological complications and lower rehabilitation time and costs (15–17). Dynamic loading may provide a mechanical stimulus to bone formation. Bone homeostasis is very linked to mechanical stimulation that can ultimately induce bone formation or bone resorption. For example, dynamic loading at low magnitude and high frequency has shown to favor the osteogenesis of alveolar bone (19). This concept has been studied extensively in the mandibular condyles. Low-magnitude, high-frequency dynamic loading can have a positive effect on condylar cartilage and endochondral bone formation *in vivo*. This effect has the potential to be used as a treatment for regenerating condylar cartilage and to enhance the effect of orthopedic appliances on mandibular growth (19, 20). The question would be whether mechanical stimulation by implant-supported prosthesis could favor the osteogenesis of severely atrophied mandible. This case series provide data on the alveolar bone changes in a severely atrophic mandible that has been treated with implant supported prosthesis under progressive loading protocol.

## Material and methods

We have conducted a retrospective case series. This retrospective study reported on 3 patients that attended the clinic with completely edentulous mandible seeking prosthetic treatment. Relevant medical data were collected from patient records. The cause of tooth loss was unknown. All patients were users of conventional complete denture and had extremely atrophic mandibles. The inferior alveolar nerve was either located in the submucosa or having 1–2 mm of bone above. The patient perspective (quality of life) was not recorded.

Implant insertion: In all patients, 4 or 5 implants were placed between foramina and all implants were anchored in the basal cortical bone (bicortically stabilized).

For that, low-speed drilling (125 rpm) without irrigation was employed (biological drilling) (21) to prepare the implant sites. The drilling sequence for each implant was individualised according to implant length, diameter and bone density at the insertion site (21). New set of drills (BTI Biotechnology Institute, Vitoria, Spain) were used in each patient to decrease the trauma to the alveolar bone. Before implant insertion, plasma rich in growth factors (PRGF) was used to irrigate the implant surface and create a bioactive zone to promote osseointegration (21). The implant insertion was performed with the surgical motor at a torque of 25 Ncm, then it was accomplished manually with a calibrated torque wrench. The final insertion torque was annotated in the patients' records. PRGF was prepared with a commercial disposable kit (KMU 15, BTI Biotechnology Institute, Vitoria, Spain) following the manufacturer instructions. After blood centrifugation, the plasma column was fraction into F2 which is the first 2 ml of plasma column just above the buffy coat and F1 which is the rest of the plasma column. F2 was activated with calcium ions (PRGF activator) and used before implant insertion.

Loading protocol: An intermediate definitive abutment (Multi-Im®) was connected to the dental implants and tightened at 25 Ncm. Impression making was performed using Impregum Penta^™^ (3M España, S.A.) and impression copings for the intermediate abutments. The implants were loaded immediately in all three patients by a fixed provisional resin prosthesis with a metal bar structure. Minimal or no cantilever extension was included in the prosthesis. During the progressive loading a fixed screw-retained resin denture was delivered. The length of the cantilever extension was adapted to growth of the residual alveolar bone at the mandible body. This had 3 stages: three months after immediate loading (stage 1), evidence of vertical bone growth (stage 2) and progression of vertical bone growth (stage 3).

Follow-up: The remaining control visits were scheduled every six months and panoramic radiographs were obtained. When panoramic radiographs showed evidence of bone thickening, CBCT was obtained to assess alveolar bone height using the BTI scan software.

**Figure 1:**
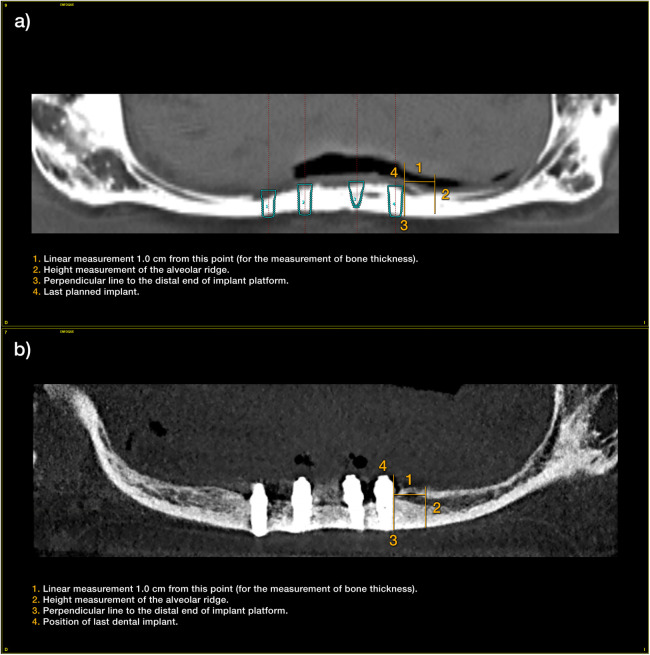
**(a)** Measurements taken at the initial cone-beam to estimate the residual bone height of the mandible since the last planned implant. **(b)** Measurements at the end of follow-up period.

Patient-related variables were age, sex, and medical history. Implant-related variables were anatomical location, implant dimensions, type of loading, implant survival, changes in the marginal bone level and radiographic measurements. Changes in the alveolar bone height were assessed at the region expanding 1 cm posteriorly to the last implant using CBCT scan, basal and after a period in which slight bone thickening is observed around the dental implants (between six months and one year after loading) (Figure 1). This load is maintained until growth of the body of the mandible is observed (between two and three years). Once the implants have been loaded and the prosthesis verified to be functioning properly, the cantilever and lever generated by the prosthesis were increased. This is done by means of a cone-beam control, which is performed at each stage of the prosthesis change and at the end of the follow-up (Figure 1).

## Results

**Figure 2:**
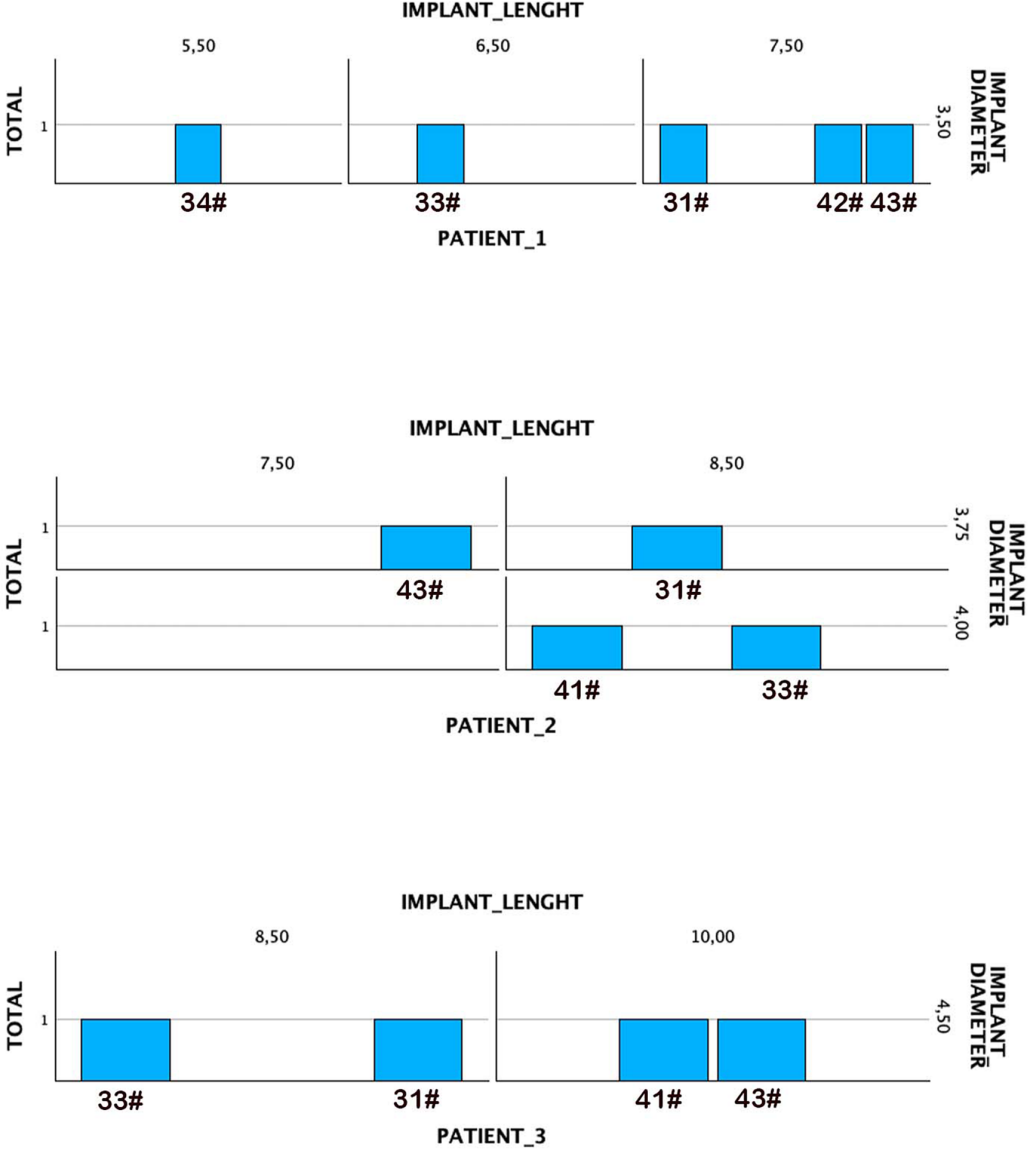
Diameters and lengths of the implants included in the study.

This case series described 3 patients where 13 implants were placed to support fixed implant-supported prostheses. The three patients were female. Their age was 64, 69 and 76 years at the time of surgery. None of the patients were smokers and the main systemics pathologies were: hypertension in two of the three patients (on treatment with beta-blockers), type II diabetes in one of the patients (in treatment with diet only) and hypercholesterolemia in one of the patients (on treatment with simvastatin). In no case was there any previous surgical pathology of interest or diseases requiring prolonged medical treatment. In two of the cases, four parallel implants were inserted, while in the third case, five parallel implants were placed. The positions of the implants corresponded to the canines and central incisors. In the case of five implants, a central para-symphyseal implant was additionally inserted. The diameters of the implants included in the study ranged from 3.5 to 4.5 mm, with the most frequent diameter being 3.5 mm. The lengths of the implants ranged from 5.5 mm to 10 mm, with 8.5 mm being the most frequent (38.5% of the cases). Figure 2 describes the implant dimensions for each patient. The mean torque of the implants included in the study was 68 Ncm (±12), with a range between 45 and 60 Ncm.

**Figure 3:**
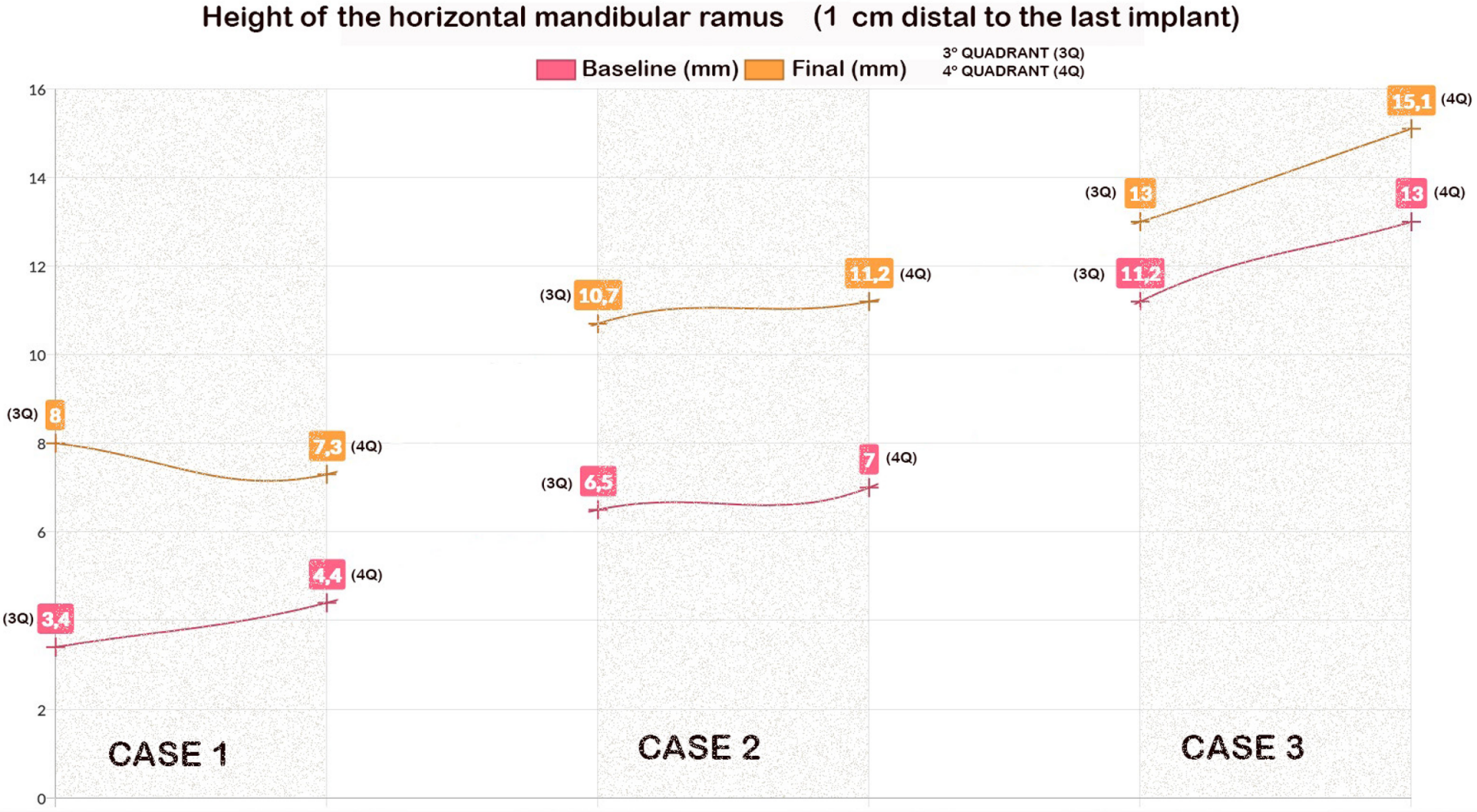
The height of the mandibular bone body in the edentulous section (1 cm linear from the last implant) for each quadrant and patient.

**Figure 4:**
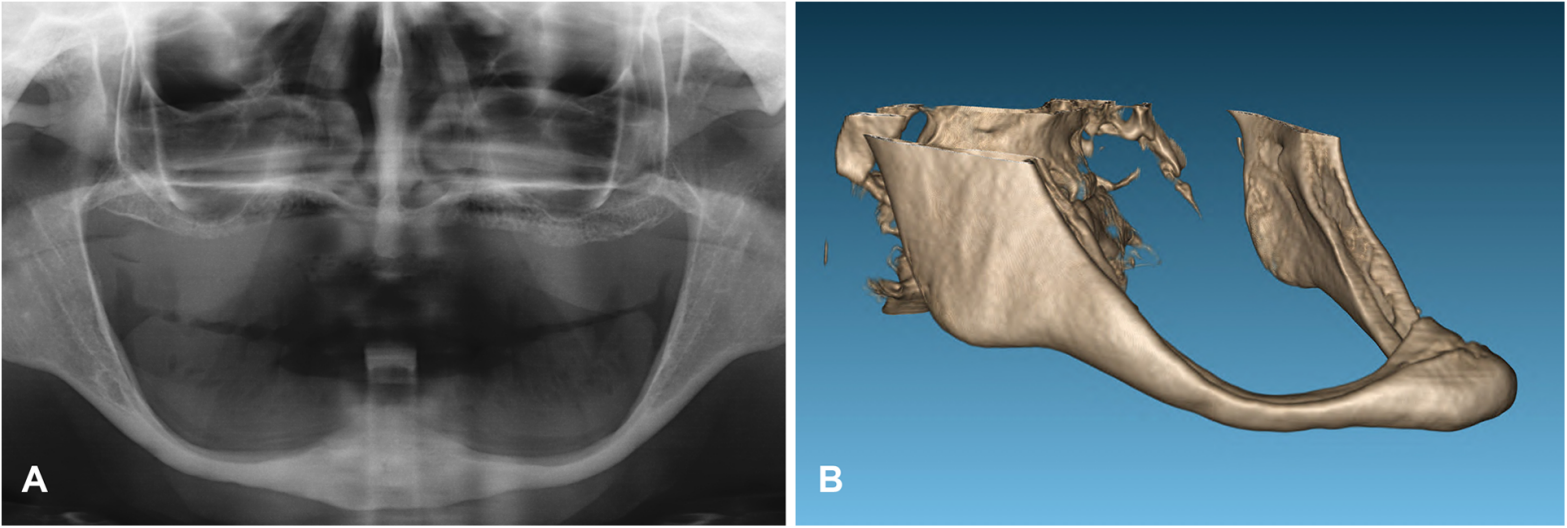
The initial x-ray **(A)** and three-dimensional reconstruction of the cone-beam of the mandible in the first case revealed significant bone resorption of the mandibular body **(B)**.

**Figure 5:**
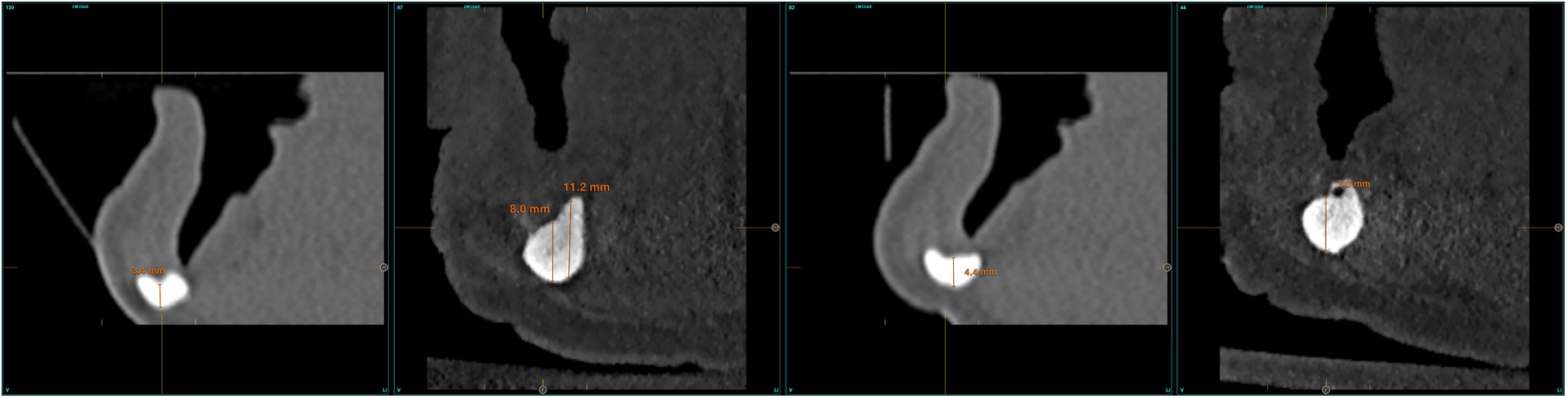
A comparison of the initial and final cone-beam images in the patient reveals the extent of the growth achieved at the level of the mandibular body. This growth has resulted in the regeneration of the inferior dental nerve canal.

The completely edentulous maxilla was rehabilitated with implant-supported fixed prosthesis in all patients. The patients were followed for 17, 19 and 20 years after implants insertion. The patient's assessment with cone-beam CT scan revealed that, in two patients, the dental nerve was running submucosally posterior to the dental foramen. In the third patient, only 1 mm of bone (bilaterally) was available above the inferior alveolar nerve (Figures 3–5).

At stage 1, the cantilever extension measured 4.1 ± 0.9 mm in the third quadrant and 4.1 ± 1.67 mm in the fourth quadrant (Table 1). Stage 2 was observed at 2 years of loading in the first patient, 3 years in the second patient and 5 years in the third patient. At this stage the cantilever extensions were increased to 6.5 ± 1.3 mm in the third quadrant and 8.1 ± 2.6 mm in the fourth quadrant (Table 1). Stage 3 was observed at 8 years after loading in the first patient, 5 years in the second patient, and 7 years in the third patient. At this stage, the definitive prosthesis was delivered. The cantilever extensions were 11.2 ± 3.2 mm in the third quadrant and 12.3 ± 5.1 mm in the fourth quadrant (Table 1). The definitive prosthesis was metal-reinforced resin and screw-retained.

**Table 1:** The evolution in the cantilever extension during the different stages of the follow-up.

	Cantilever extension (mm) (Mean ± standard deviation)		
	Stage 1	Stage 2	Stage 3
Third quadrant	4.1 ± 0.9	6.5 ± 1.3	11.2 ± 3.2
Fourth quadrant	4.1 ± 1.67	8.1 ± 2.6	12.3 ± 5.1

The mean mandibular body height at 1.0 cm distal to the last implant was 7.0 ± 3.9 mm in the third quadrant and 8.13 ± 4,41 mm in the fourth quadrant. The mean height at the last follow-up was 11.0 ± 3.2 mm (±3.21) in the third quadrant and 11.20 ± 4.4 mm in the fourth quadrant. Figure 3 shows these measurements for each quadrant and patient. The mean vertical alveolar bone gain was 4.0 ± 0.7 in the third quadrant and 3.1 ± 1.0 mm in the fourth quadrant (Figure 3).

At the last follow-up and due to the stimulation of vertical alveolar bone growth, a neoformation of the mandibular canal (bone height of 1–2 mm above the nerve) was observed in 2 patients where the inferior alveolar nerve was running submucosally. In the third patient, a thickening of the residual bone above the nerve was observed. The residual alveolar bone height was increased from 1 mm to 2.5 mm.

Figures 4–11 show the cases included in the study.

**Figure 6:**
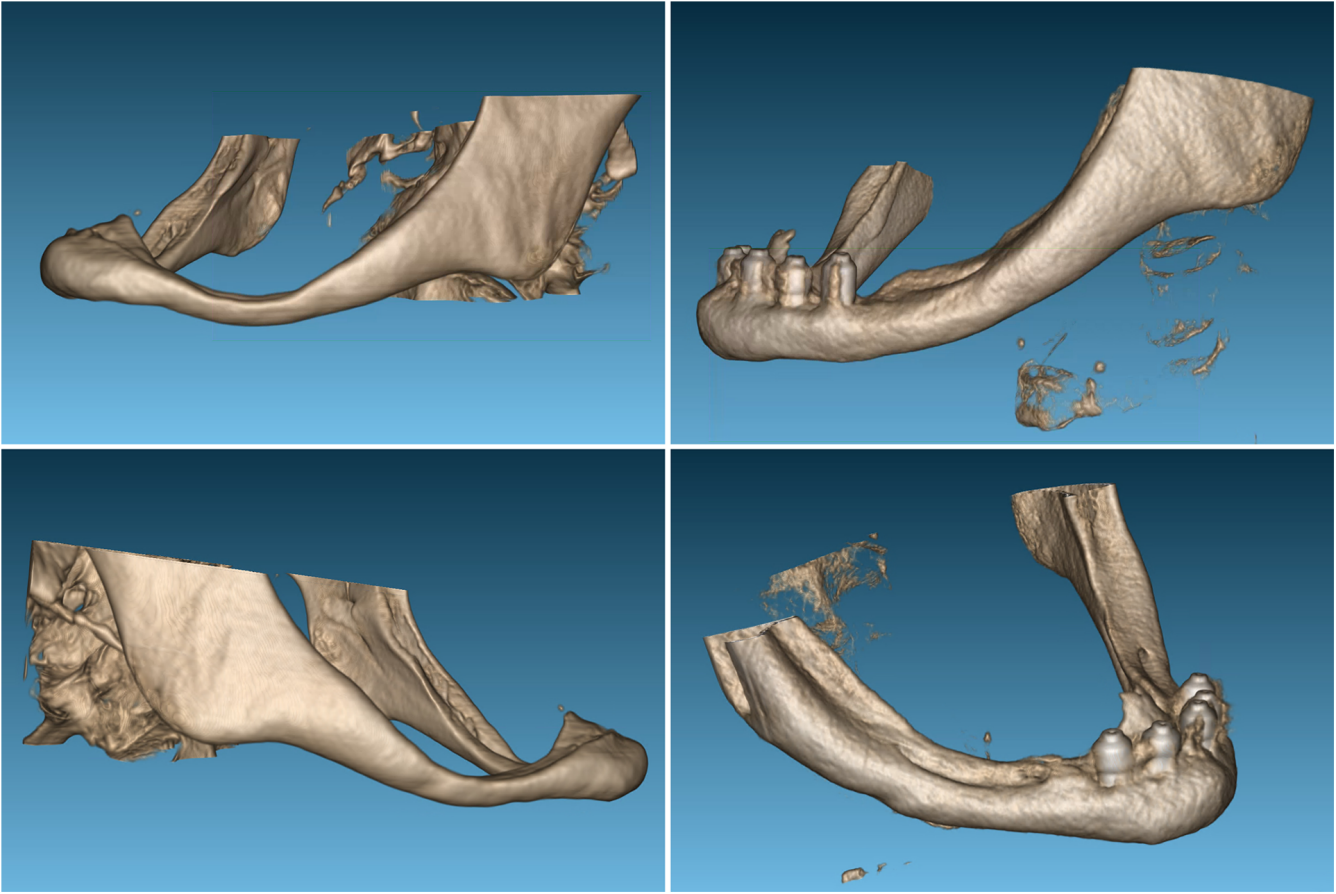
A comparison of the three-dimensional reconstructions from the cone-beam imaging reveals a clear thickening of the area corresponding to the mandibular body studied in the first case.

**Figure 7:**
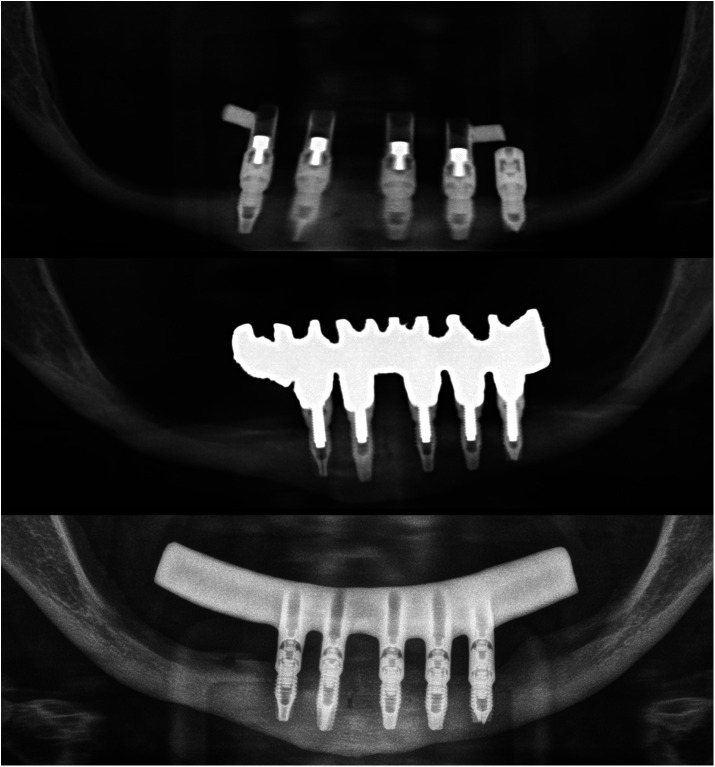
The initial prosthesis was loaded immediately and had minimal cantilever. The second set of prostheses had a wider mandibular body and was placed at the end of the follow-up at 20 years, with a full cantilever in the first case. Five implants were selected with the most distal third quadrant, with slight displacement of the dental nerve that was submucosal.

**Figure 8:**
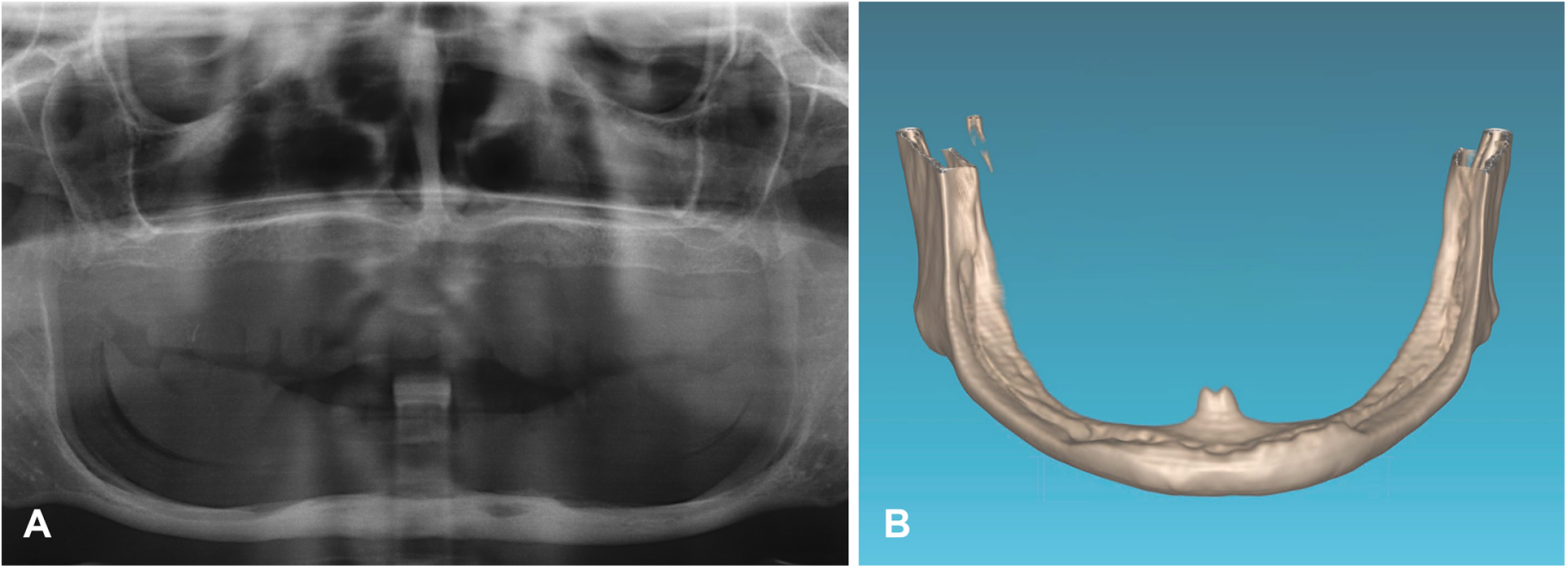
The initial x-ray **(A)** and three-dimensional reconstruction of the mandible in the second case reveals the dental nerve's path through the entire submucous mandibular body, without a protective bony roof **(B)**.

**Figure 9:**
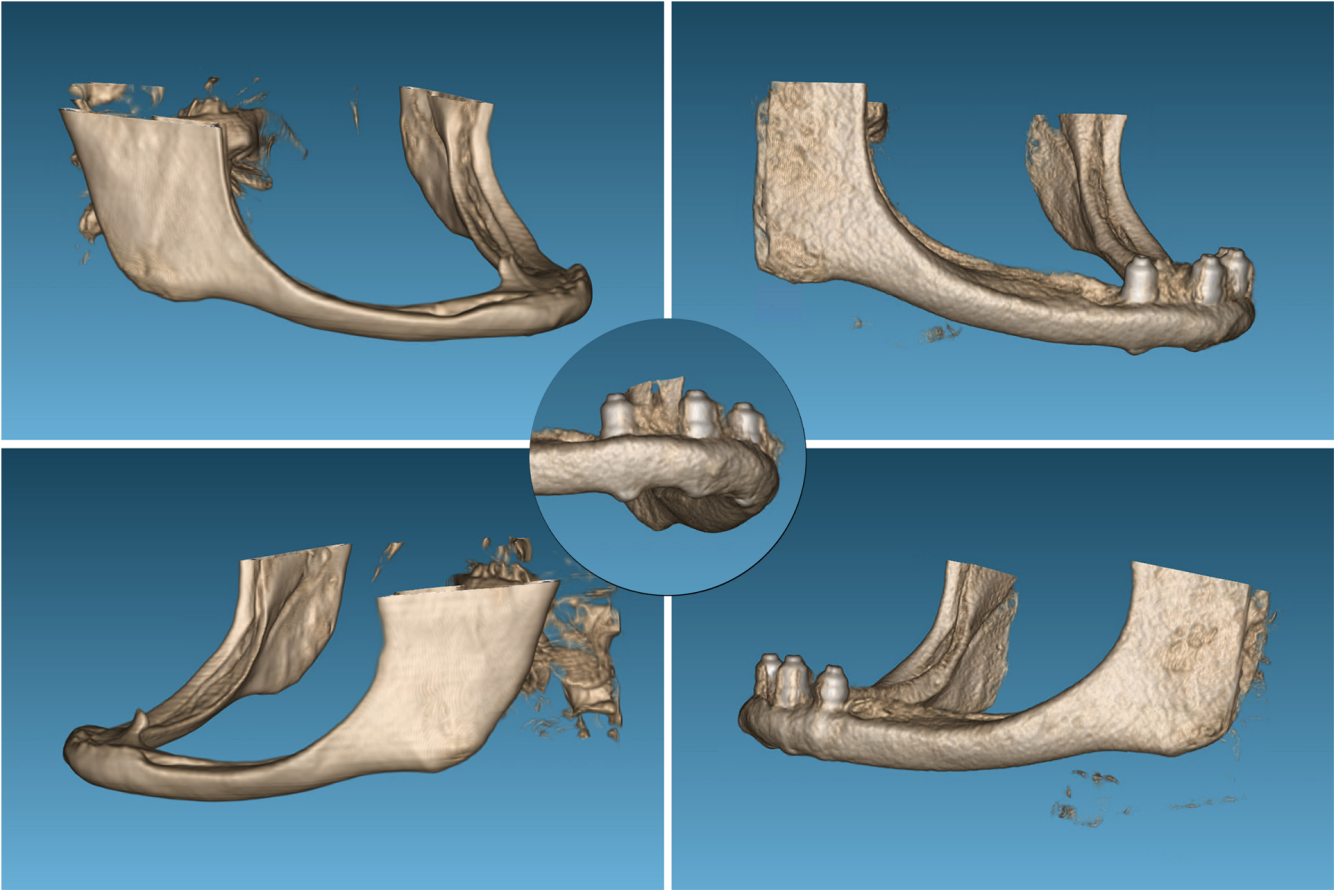
Three-dimensional reconstruction of the mandible with the growth achieved at the level of the mandibular body, in this case even apically on the implants, as evidenced by the detailed image.

**Figure 10:**
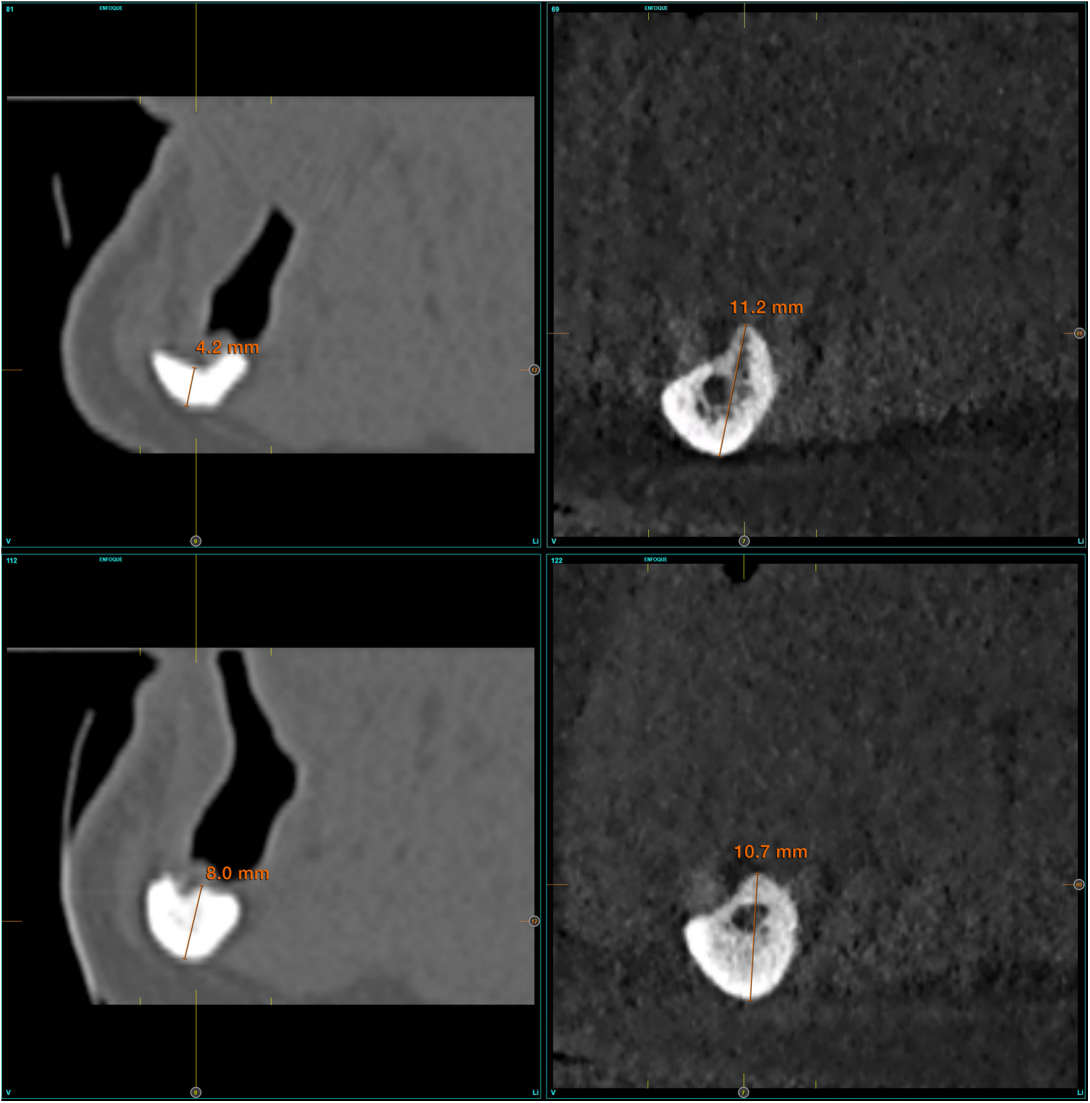
Formation of the new dental nerve canal in the third and fourth quadrant of patient n°2, as seen in the images at 17 years at the conclusion of the follow up period.

**Figure 11:**
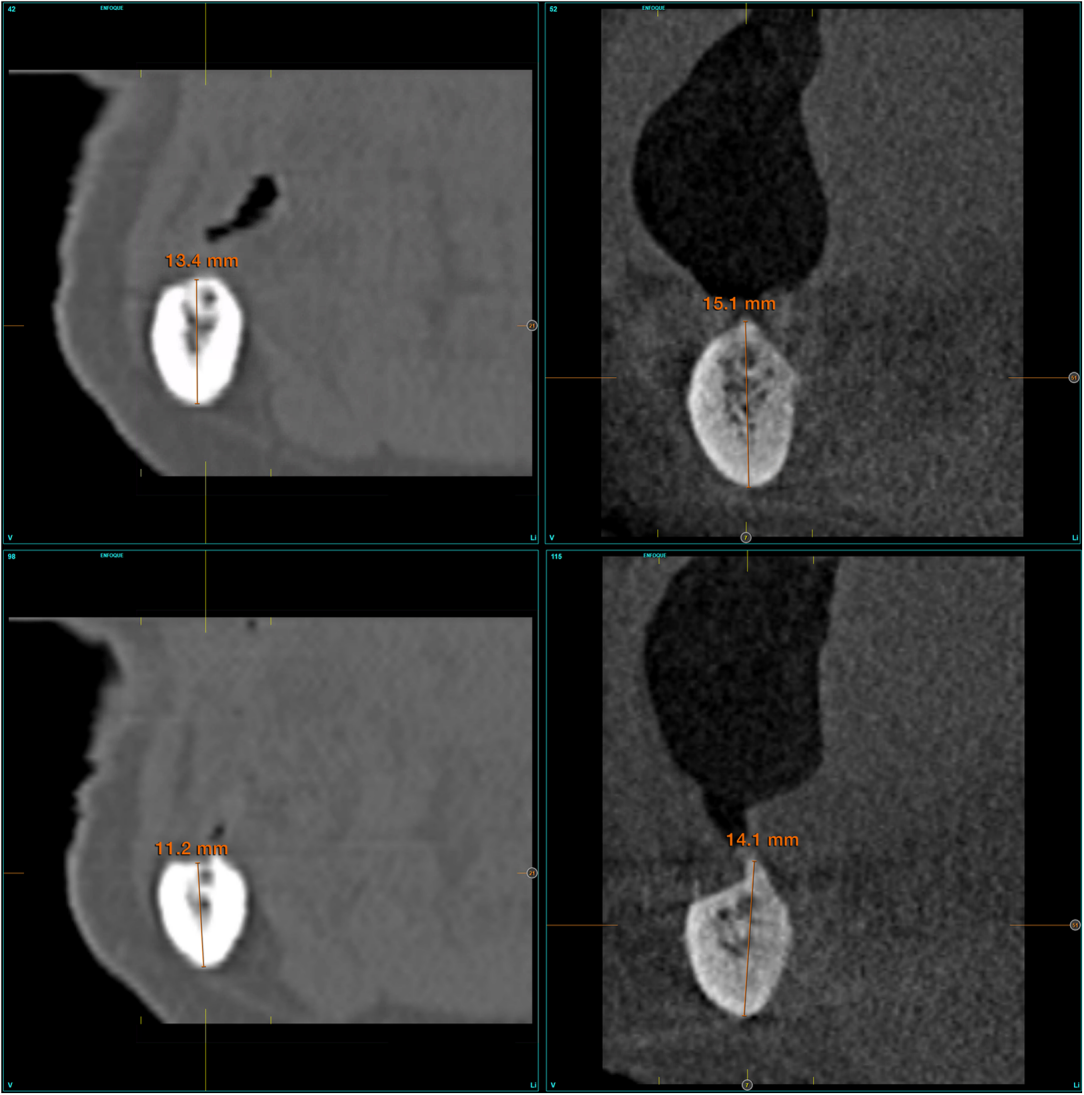
In the third case, the pre-existing dental canal exhibited an increase in thickness from 1 to 3 mm.

## Discussion

Mechanical stimulation of bone is a critical environmental factor that commonly generates tensile stress, compressive stress and fluid shear stress (22).

The merely use of dental implants does not necessarily result in a biomechanical load that optimize bone remodeling. The implant distribution, the prosthesis type and the implant dimensions are influencing factors (23–27).

Implantology, both in its surgical and prosthetic facets, is in a state of constant evolution. Modifications are being made to the procedures to achieve the best long-term results with the minimum intervention (27, 28). Extreme atrophies, such as those depicted in this study, are challenging to resolve. At that time, there have been no extra-short or ultra-short implants available (29–32). Vertical bone growth around dental implants has been described in the international literature as a surgical technique that is intentionally performed to achieve 1–2 mm of bone apposition on the surface of an implant that has been placed supra-crestal to all or part of the implant (33, 34). In the cases presented in this work, no specific surgical technique was used to increase the residual bone volume of the mandible, but vertical bone growth has been observed.

The phenomenon of active and continuous bone remodeling in the presence of physiological loads is well documented, stimulating the proliferation of cell lines that should generate active bone replacement. In general, stresses exceeding those typically encountered in everyday life (between 0.1% and 0.25%) but below the elastic limit of the bone result in bone strengthening (35–37). The stress received at a given point in the bone is transmitted through the osteocyte channels, resulting in the amplification of the mechanical signal at a distance. This process activates the osteoblasts and osteoclasts that reside on the bone surface (35, 38–41). In implant dentistry, research is being conducted into the phenomenon of osseointegration and the subsequent bone neoformation. Several factors have been identified as key to the genesis of new bone matrix, including the material and design of the implant, the characteristics of its surface, and its anatomical location (36, 37).

When load is included as an additional factor, it is observed that progressive loading generates a greater bone-to-implant contact (BIC) and a greater density in the bone between the implant threads, with statistically significant differences (42–46). The work published by Romanos et al. on monkeys highlights the importance of progressive loading of implants in bone neoformation and the benefit of provisional prostheses that can later be changed, generating a phenomenon of slow adaptation (42, 43). The cases presented in this study indicate that a progressive loading of the implants with increasing tension and cantilever extension over time may have been a key factor in vertical growth. Furthermore, bone growth in the mandibular body was accompanied by regeneration of the dental nerve canal, which did not exist before in two of the three patients. These two facts have not been previously reported in the scientific literature. Molecular response circuits have been described in bone cells with plasticity like those generated in the nervous system, which enables this “memory” to occur (44, 47).

Regarding the design of the prosthesis and its cantilever, the progressive increase in its length has been linked to changes in the alveolar bone. The distal cantilever generates an increase in tension in the last implant, which can dissipate through the mandible and towards the area posterior to the implant. This can therefore produce an increase in the stimulation of the bone cells included in that area. The distribution of stress within the bone has been validated by finite element modeling conducted by our research group in the context of investigating the impact of distal cantilevers in edentulous mandibles, as demonstrated by the case series presented here (42, 43). It is not feasible to construct the cantilever from the outset with the final length achieved in this instance of severe atrophy. This is because the stress received by the bone would exceed the threshold of stimulation, leading to bone resorption and because there is a risk of mandibular fracture associated with the stress (48, 49). The characteristics of the bone where the implants are placed and where a prosthesis with cantilever is made are crucial in determining the distribution of stresses within the bone and the potential effects that can be generated in it. As stated by Chakraborty et al. (42), the presence of a distal cantilever in the prosthesis structure is the most influential design factor on bone stresses. These stresses vary widely, ranging from 28 to 32% depending on the density and volume of the bone. Therefore, gradual monitored augmentation should be the recommended approach in these situations to ensure the long-term success of the treatment and to avoid complications due to bone stress.

In the three patients treated in this work, we have considered fixed implant prostheses as the best therapeutic option, discarding overdentures. This decision was based mainly on the long-term comfort of the patient, the age of the patients and their life expectancy, on the maintenance of the crestal bone around the implants, and on the working philosophy of our study group (50–52).

This study suffers from the limited sample size and the limitations of the retrospective design. However, it has reported novel observations in relation to vertical bone growth in severely atrophic mandible after being treated with implant-supported prosthesis. It should also be noted that when the treatment with implants was performed on these patients (between 17 and 20 years ago) the concept of dynamic loading had not been identified or studied yet, so the loading process performed on the implants and the progressive increase of the load was also something new at the time it was carried out. The long-term follow-up give a strength to the observation related to a continuous vertical bone gain. The linear measurements of marginal bone dynamics have been performed on CBCT images, however, the beam hardening in the vicinity of the implant would be a source of error to the measurements as it limits the precise identification of the marginal bone level. Furthermore, the 3D reconstruction of CBCT images is subjected to errors and artifacts due to the lack of reproducibility of the CT numbers and thus the reproducibility of the 3D reconstruction of CBCT images.

## Conclusions

Implant-supported prosthesis and progressive loading have resulted in vertical bone growth in a series of patients with extreme atrophy of the mandible. The long-term follow-up indicated alveolar bone growth resulting n the thickening of the mandibular body and the creation of the absent mandibular canal.

We should select both the patients and the professionals who treat this type of severe atrophy, since the insertion of implants in bones with these characteristics can generate complications such as mandibular fractures. Therefore, we recommend a detailed analysis of the case and the performance of the surgery by expert surgeons to obtain the best results.

## Data Availability

The raw data supporting the conclusions of this article will be made available by the authors, without undue reservation.
